# Pre-Conditioning with IFN-γ and Hypoxia Enhances the Angiogenic Potential of iPSC-Derived MSC Secretome

**DOI:** 10.3390/cells11060988

**Published:** 2022-03-14

**Authors:** Suya Wang, Felix Umrath, Wanjing Cen, António José Salgado, Siegmar Reinert, Dorothea Alexander

**Affiliations:** 1 Department of Oral and Maxillofacial Surgery, University Hospital Tübingen, 72076 Tübingen, Germany; suya.wang@student.uni-tuebingen.de (S.W.); felix.umrath@med.uni-tuebingen.de (F.U.); cenwanjingwj@gmail.com (W.C.); siegmar.reinert@med.uni-tuebingen.de (S.R.); 2 Life and Health Sciences Research Institute (ICVS), School of Medicine, University of Minho, Campus de Gualtar, 4710-057 Braga, Portugal; asalgado@med.uminho.pt; 3 ICVS/3B’s–PT Government Associate Laboratory, University of Minho, 4710-057 Braga, Portugal

**Keywords:** iPSC-derived MSCs, iMSC secretome, pre-conditioning, angiogenesis, IFN-γ, hypoxia, potentiation of iMSC efficacy

## Abstract

Induced pluripotent stem cell (iPSC) derived mesenchymal stem cells (iMSCs) represent a promising source of progenitor cells for approaches in the field of bone regeneration. Bone formation is a multi-step process in which osteogenesis and angiogenesis are both involved. Many reports show that the secretome of mesenchymal stromal stem cells (MSCs) influences the microenvironment upon injury, promoting cytoprotection, angiogenesis, and tissue repair of the damaged area. However, the effects of iPSC-derived MSCs secretome on angiogenesis have seldom been investigated. In the present study, the angiogenic properties of IFN-γ pre-conditioned iMSC secretomes were analyzed. We detected a higher expression of the pro-angiogenic genes and proteins of iMSCs and their secretome under IFN-γ and hypoxic stimulation (IFN-H). Tube formation and wound healing assays revealed a higher angiogenic potential of HUVECs in the presence of IFN-γ conditioned iMSC secretome. Sprouting assays demonstrated that within Coll/HA scaffolds, HUVECs spheroids formed significantly more and longer sprouts in the presence of IFN-γ conditioned iMSC secretome. Through gene expression analyses, pro-angiogenic genes (FLT-1, KDR, MET, TIMP-1, HIF-1α, IL-8, and VCAM-1) in HUVECs showed a significant up-regulation and down-regulation of two anti-angiogenic genes (TIMP-4 and IGFBP-1) compared to the data obtained in the other groups. Our results demonstrate that the iMSC secretome, pre-conditioned under inflammatory and hypoxic conditions, induced the highest angiogenic properties of HUVECs. We conclude that pre-activated iMSCs enhance their efficacy and represent a suitable cell source for collagen/hydroxyapatite with angiogenic properties.

## 1. Introduction

The reconstruction of large tissue defects is one of the main challenges in the field of oral and maxillofacial surgery. Despite some significant limitations, including donor site morbidity, restricted availability, and poor bone quality [1], autologous grafting has remained the gold standard for bone over the years [2]. In order to overcome these drawbacks, tissue engineering with stem cells, signal molecules, and scaffolds has attracted attention in the area of regenerative medicine [3,4]. This therapeutic procedure benefits from the regenerative capacity of the human body through the application of adult stem cells in combination with optimized synthetic materials [5]. As oxygen and nutrient supply is essential for survival within the graft, the use of suitable cells to promote angiogenesis and to recruit endothelial cells has gained in importance.

Mesenchymal stem cells (MSCs) have been shown to be a promising cell candidate for cell-based therapy [6]. MSCs have attracted the interest of clinician-scientists not only because of the low immunogenicity and tissue regenerative properties that could enable their use in allogeneic settings [7], but also for their anti-tumorigenic, anti-fibrotic, anti-apoptotic, anti-inflammatory, pro-angiogenic, neuro-protective, anti-bacterial, and chemo-attractive effects [8,9,10]. Recently, it has become evident that the functional benefits exerted by MSCs upon transplantation are due to the release of paracrine factors and biologically relevant molecules to the neighboring diseased or injured tissue, referred to as the MSCs secretome [11,12,13]. The secretome of MSCs influences the microenvironment upon injury, promoting cytoprotection, angiogenesis, and tissue repair at the damaged area [14].

MSCs differentiated from induced pluripotent stem cells (iPSCs), called induced mesenchymal stem cell-like cells (iMSCs), represent an alternative to primary MSCs isolated from different tissues [1]. Due to the limited availability, in vitro proliferation capacity, and differentiation potential of primary MSCs, impeding their application in the clinical routine [15], iMSCs have been identified as a promising source of transplantable donor cells with similar capacities.

A variety of studies have demonstrated that MSCs and iMSCs have comparable properties either in their morphology, marker expression, differentiation potential, or immunomodulatory properties [16,17]. The advantages of the use of iMSCs are that they have been characterized as rejuvenated MSCs [18,19,20] and that they show no risk of tumor formation as they do not express oncogenic pluripotency-associated genes such as OCT4 [21]. In addition, iMSCs outperformed native MSCs in the treatment of multiple sclerosis in a rodent model [22]. Another animal study demonstrated that iMSCs could successfully improve in vivo bone regeneration by their direct differentiation into bone cells and by the recruitment of host cells in a radial defect model in mice [23].

In previous studies, our lab succeeded in the generation of iPSCs from jaw periosteal cells (JPCs) and the resulting differentiation of iPSCs to iMSCs [24,25]. We provided evidence for tri-lineage differentiation of generated iMSCs compared to that of the parental JPCs by histologic staining and gene expression patterns. Furthermore, we gave information about their morphology and telomere lengths, about their proliferative and mitochondrial activities, and about cellular senescence compared to that detected in parental JPCs.

Angiogenesis and bone formation are coupled processes during skeletal development and fracture healing [26]. New blood vessels bring oxygen and nutrients to the metabolically highly active regenerating callus, and serve as a route for inflammatory cells, cartilage, and bone precursor cells to reach the injury site [27]. It has been suggested that the MSC secretome induces angiogenesis [28].

To the best of our knowledge, the present study is the first study examining the influence of iMSCs secretomes on angiogenesis. The aim of our study was to compare the angiogenic potential of the secretome from differently pre-conditioned iMSCs in order to find a suitable cell source with pro-angiogenic capacities for the generation of impactful bone tissue engineering constructs.

## 2. Materials and Methods

### 2.1. Cell Culture

iMSCs derived from three donors were grown in hPL10-medium (DMEM/F12 (Gibco) + 10% human platelet lysate (hPL; PL BioScience GmbH, Aachen, Germany), 100 U/mL penicillin-streptomycin (Pen-Strep; Lonza, Basel, Switzerland), and 2.5 μg/mL amphotericin B (Biochrom, Berlin, Germany). Passages 5-6 iMSCs were used for this study [25].

### 2.2. iMSC Stimulation and Secretome Collection

**Figure 1:**
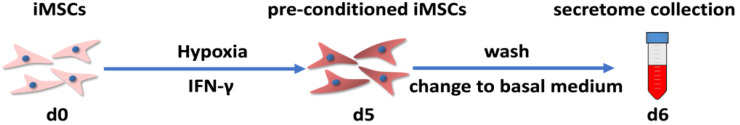
Timeline of IFN-γ stimulation and secretome collection: iMSCs were seeded into T-175 cell culture flasks (d0) and were stimulated with IFN-γ under hypoxic condition for 5 days (d5). Cells were washed and medium change to basal medium followed. After 24 h culture with basal medium, iMSCs secretomes were collected at day 6 (d6).

First, 2.3 × 10^6^ iMSCs were seeded into T-175 cell culture flasks (Greiner Bio-One GmbH, Frickenhausen, Germany). For pro-inflammatory conditions (Figure 1), iMSCs were stimulated with IFN-γ (200 ng/mL, Sigma-Aldrich, St. Louis, MO, USA) for 5 days under normoxia (21% O_2_, 5% CO_2_, IFN-N group) and hypoxia (5% O_2_, 5% CO_2_, IFN-H group).

As control groups, iMSCs were cultured only in both normoxic and hypoxic conditions without IFN-γ stimulation. After 5 days, the medium was removed, the cells were washed three times with 10 mL PBS and 10 mL basal medium (DMEM/F12, 100 U/mL penicillin-streptomycin, and 2.5 μg/mL amphotericin B), then subsequently starved in 37 mL basal medium without hPL supplementation. After 24 h, 37 mL of iMSCs secretome was collected in 50 mL tubes (Greiner Bio-One GmbH, Frickenhausen, Germany). After centrifuging these tubes at 600 g for 7 min to discard the cell debris, 34 mL of supernatant was recollected in fresh 50 mL tubes. After rapid freezing in liquid nitrogen, the secretome was stored at −80 °C in a freezer until the process of secretome enrichment.

### 2.3. Flow Cytometric Analyses of HLA-I and HLA-II Expression of iMSCs

As the parental JPCs were shown to be very sensitive to IFN-γ stimulation, we determined the optimal duration of IFN-γ treatment in pre-experiments. JPCs as well as other MSCs respond to IFN-γ through the up-regulation of their HLA-II expression. We treated iMSCs with IFN-γ for 3, 5, and 7 days, and detected the highest HLA-II response at day 5. Therefore, IFN-γ stimulation for 5 days was chosen for all of the experiments performed for this study. Stimulated iMSCs and cells from the control groups were collected for flow cytometric analyses. Then, 2 × 10^5^ cells per sample were incubated in 20 µL blocking buffer (PBS, 0.1% BSA, 1 mg/mL sodium azide (Sigma-Aldrich) and 10% Gamunex (human immune globulin solution, Talecris Biotherapeutics, Frankfurt, Germany) for 15 min on ice. Then, the cells were incubated on ice with a FACS buffer (DPBS, 0.1% BSA, and 0.1% sodium azide) as well as mouse APC-conjugated anti-HLA-I and anti-HLA-II antibodies (MACS Miltenyi Biotec, Bergisch Gladbach, Germany) for 20 min in the dark. For the isotype controls, APC-labeled IgG2a antibodies (Biolegend, San Diego, CA, USA) were used. For the analysis of the iMSC and iPSC surface marker expression, the APC- and PE-labeled antibodies listed in Table 1 were used. Representative histograms are shown in Supplementary Figure S1. After two washing steps with a FACS buffer, the cell pellets were resuspended in 200 μL FACS buffer and were analyzed by flow cytometry using the Guava easyCyte 6HT-2L (Merck Millipore, Billerica, MA, USA). For data evaluation, the guavaSoft 2.2.3 (InCyte 2.2.2, Luminex Corporation, Chicago, IL, USA) software was used.

### 2.4. Secretome Enrichment

According to the instruction of Vivaspin^®^ 20 centrifugal concentrators (5 kDa cut off, Sartorius, Goettingen, Germany), iMSC secretome was concentrated to 100-fold. First, 14 mL of iMSC supernatant was pipetted into the Vivaspin tubes and centrifuged at 6000× *g* (6831 rpm). After ca. 2 h of centrifugation, the volume from the upper compartment of the tube was checked and further volume was filled for secretome enrichment until the total volume of the added supernatant reached 34 mL (14 mL + 10 mL + 10 mL). After a total centrifugation time of 9 h, less than 340 µL of the concentrated iMSC secretome were collected and filled to exactly 340 µL with basal medium (DMEM/F12, 100 U/mL penicillin-streptomycin, and 2.5 μg/mL amphotericin B). After the enrichment of the secretomes from three different donors, those obtained under the same condition were mixed completely in order to minimize the interindividual variations and to obtain more reliability of the data. The optimal iMSC secretome concentration was determined by dose response kinetics. Therefore, tube formation assays were performed in the presence of 1×, 5×, and 10× concentrated secretomes. As illustrated in Supplementary Figure S2, 10× secretomes showed the highest angiogenic effect. Based on this result from the pre-experiments, all other experiments were performed with mixed 10-fold concentrated iMSC secretomes.

### 2.5. Detection of IL-8 and VEGF-A Protein Concentrations in iMSC Secretomes by Enzyme-Linked Immunosorbent Assay (ELISA)

The secretomes from different conditions were collected and IL-8 and VEGF-A protein secretions were quantified using the human IL-8 ELISA kit II (Invitrogen, Thermo Fisher, Darmstadt, Germany) and the human VEGF ELISA kit (R&D Systems, Minneapolis, USA) according to the manufacturer’s instructions. All measurements were performed in duplicate. ELISA plates were read immediately with a microplate ELISA reader (BioTek, Friedrichshall, Germany) at a wavelength of 450 nm. IL-8 and VEGF concentrations were quantified using a standard curve of known concentrations. The lowest detection limit was in the range of 9–15.63 pg/mL.

### 2.6. Cell Culture of Human Umbilical Vein Endothelial Cells

Human umbilical vein endothelial cells (HUVECs) were purchased from PromoCell (Heidelberg, Germany) and cultured in endothelial cell growth medium 2 (EGM-2 kit, PromoCell, Heidelberg, Germany) with 1% amphotericin B and penicillin/streptomycin (Biochrom, Berlin, Germany) at 37 °C and 5% CO_2_. Cells of passages 5–7 were used for all experiments and medium change was performed three times per week.

### 2.7. Endothelial Tube Formation

Tube formation assays were performed with HUVECs using a method adapted from Wang and co-authors [29]. Briefly, 100 μL/well of GeltrexTM LDEV-free reduced growth factor basement membrane matrix (Invitrogen/Thermo Fisher Scientific, Waltham, MA, USA) was incubated in a 24 well plate for 45 min at 37 °C for matrix gel polymerization. Then, 5 × 10^4^ HUVECs were seeded onto GeltrexTM matrices and cultured for 8 h with EGM-2 medium containing all the supplements (+GF, positive group) and EGM-2 medium containing 10× secretome of different conditions (IFN-H, IFN-N, Co-H, and Co-N). EGM-2 medium without growth factors served as the negative control (-GF). After 8 h of cultivation, 1 μM calcein-AM dye (Invitrogen/Thermo Fisher Scientific, Waltham, MA, USA) was added to the plate and incubated for 20 min. Fluorescence images were captured from at least three wells per culture condition at a 1.25× magnification using the Axio Observer Z1 fluorescence microscope (Zeiss, Oberkochen, Germany). Network branches, meshes, and nodes were counted from the collected images using the Image J software, in order to quantify the angiogenic network formation.

### 2.8. Detection of Wound Closure by Using the Endothelial Scratch Assay

An in vitro scratch assay was performed as described previously [30]. HUVECs were seeded in 24-well plates at a density of 1 × 10^5^. After incubation for 24 h, each colonialized well was manually scratched with a 200 μL pipette tip, washed with PBS three times, and incubated at 37 °C under different conditions (positive, IFN-H, IFN-N, Co-H, Co-N, and negative groups). Three randomly selected views along the scraped line were photographed from each well immediately after manual scratching and 8 h later with a 5× objective in a brightfield microscope. The area of the wound gap was calculated using the ImageJ software:

$$\%\;\mathrm{wound}\;\mathrm{closure}=\frac{A\left(0\right)-A\left(t\right)}{A\left(0\right)}\times 100\%$$

where A(t) is the wound area at 8 h and A(0) is its initial area. Cell migration was quantified and expressed as the average percentage of closure of the scratch area [31].

### 2.9. Spheroid Sprouting Assay in Collagen/Hydroxyapatite Scaffolds

A spheroid sprouting assay was adapted from the method previously described by Maracle and co-authors [32]. The principle of this assay is based on the sprout formation originating from aggregated and gel-embedded HUVECs. In brief, methocel solution was prepared by dissolving 6 g methylcellulose (Sigma-Aldrich, USA) in 500 mL EGM-2 medium (PromoCell, Heidelberg, Germany). The HUVECs were then harvested. A total of 8 × 10^3^ HUVECs were added to each well of a 96-well polypropylene plate (Corning, Sigma-Aldrich, USA) in 200 μL EGM-2 medium containing 20% methocel. Spheroids formed overnight at 37 °C. Then, 30 spheroids were resuspended in 300 μL of the solution for the preparation of collagen/hydroxyapatite composites. After incubation at 37 °C for 30 min for polymerization of the collagen/hydroxyapatite composites, 500 μL of the different pre-conditioned iMSCs secretomes containing 10% FBS (IFN-H, IFN-N, CO-H, and CO-N groups) were added to the wells. Sprout formation by the HUVEC spheroids was detected after 48 h of incubation.

### 2.10. Fluorescence Staining

Different groups of collagen/hydroxyapatite scaffolds containing HUVEC spheroids were washed three times with PBS and were fixed with 4% paraformaldehyde for 1 h. After cell permeabilization with PBS + 1% Triton-X100 (Sigma), the cells were washed with PBS and stained with Alexa488-Phalloidin (10 μg/mL in bovine serum albumin, Sigma-Aldrich, USA) and Hoechst 33342 (1 μg/mL, Promocell, Heidelberg, Germany) at room temperature for 1 h. After three wash steps with PBS, images were taken using an Axio Observer Z1 fluorescence microscope (Zeiss, Oberkochen, Germany) at 10× magnifications. Spheroids were quantified using the ImageJ software in order to measure the length of the sprouts and to calculate the cumulative sprout length (CSL). Data from at least 10 spheroids per experimental group were calculated.

### 2.11. RNA Isolation and Quantitative Gene Expression Analyses in iMSCs and HUVECs

The total mRNA was isolated from HUVECs using the NucleoSpin RNA kit (Macherey-Nagel, Hoerd, France) according to the manufacturer’s guidelines. After isolation, RNA was quantified using a Nanodrop micro-volume spectrophotometer (Invitrogen/Thermo Fisher Scientific, Waltham, MA, USA). Then, cDNA synthesis was performed using the LunaScript^®^ RT SuperMix kit according to the instructions of the manufacturer (New England Biolabs, Ipswich, MA, USA). To quantify the mRNA expression levels, the Applied Biosystems^®^ QuantStudio^®^ 5 Real-Time PCR System (Thermo Fisher Scientific, Waltham, MA, USA) was used. For the PCR reactions, DEPC-treated water, Luna^®^ Universal Probe qPCR Master Mix (New England Biolabs, Ipswich, MA, USA), and primer kits (FLT-1, KDR, HGF, MET, IL-8, HIF-1α, MMP-1, TIMP-1, TIMP-4, IGFBP-1, IGFBP-2, and VCAM-1) from Thermo Fisher Scientific (Waltham, MA, USA) were used for 40 amplification cycles of the target cDNA following the manufacturer’s instructions. The target gene transcript levels were normalized to those of the housekeeping gene GAPDH. X-fold induction values were calculated by the quotient of the sample and the corresponding control. All cDNA samples were analyzed in triplicate.

### 2.12. Statistical Analysis

The data of all measurements are expressed as means ± standard error of means (SEM). All statistical analyses were carried out using the GraphPad Prism software (La Jolla, CA, USA). The two-tailed Student’s t-test or one-way analysis of variance (ANOVA) for repeated measurements followed by Tukey’s multiple comparisons tests were used. Values were considered significant with a *p*-value < 0.05.

## 3. Results

### 3.1. HLA-I and HLA-II Expression by iMSCs Cultured under Pro-Inflammatory (IFN-γ) and Hypoxic/Normoxic Condition

In a previous study, we provided evidence of tri-lineage differentiation, telomere lengths, and proliferative and mitochondrial capacities of generated iMSCs, compared to the functions of the parental JPCs [25]. The MSC and iPSC marker expressions of the iMSCs used in this work are given in the Supplementary Figure S1.

**Figure 2:**
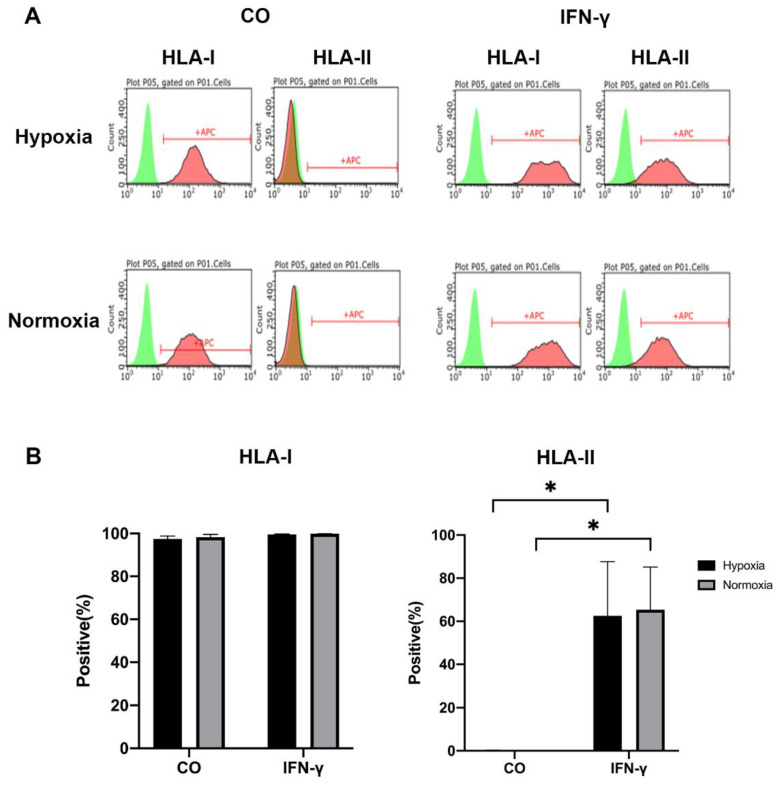
HLA-I and HLA-II expression of iMSCs of untreated (CO) and stimulated (with 200 ng/mL IFN-γ (IFN-γ groups) for 5 days under hypoxic and normoxic conditions. (**A**) Representative histograms of the surface marker expression were detected by flow cytometry. Positive cells (red), unstained control (green). (**B**) Amount of HLA-I and -II positive cells under the indicated conditions. Differences in surface marker expression were compared using two-way ANOVA (*n* = 3 patients, * *p* < 0.05).

To characterize the response of iMSCs to IFN-γ stimulation, the expression of HLA-I and HLA-II surface markers was analyzed via flow cytometry. iMSCs derived from three donors expressed high levels of HLA-I, under both hypoxic and normoxic conditions (Figure 2A). After iMSC stimulation with IFN-γ for 5 days (IFN-γ), the expression of HLA-II (Hypoxia: IFN-γ: 62.52 ± 25.21%; Normoxia: IFN-γ: 65.37 ± 19.79%) was significantly upregulated compared to that of the untreated iMSCs (CO) (Normoxia: CO: 0.12 ± 0.01%; Hypoxia: CO: 0.27 ± 0.09%) under both hypoxic and normoxic conditions (Figure 2B).

### 3.2. Gene Expression of the Pro-Angiogenic Genes IL-8 and VEGF-A by iMSCs Cultured under Pro-Inflammatory (IFN-γ) and Hypoxic/Normoxic Conditions

**Figure 3:**
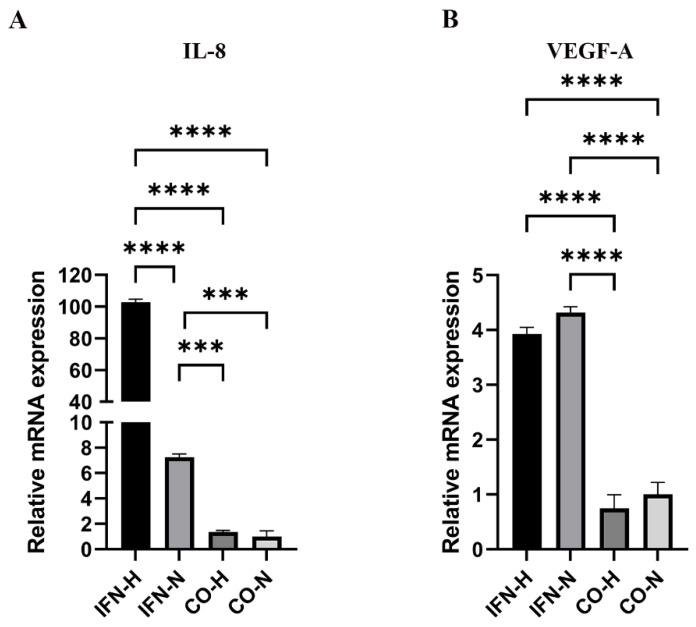
Expression of angiogenesis-related genes by iMSCs pre-conditioned with IFN-γ under hypoxic and normoxic conditions. Gene expression levels of IL-8 (**A**) and VEGF-A (**B**) in iMSCs were quantified using a Thermo Fisher Scientific PCR instrument, and ratios of the target mRNA copy numbers related to copy numbers of the housekeeping gene (GAPDH) were calculated. Gene expressions mean values ± SEM in iMSCs from the IFN-H, IFN-N, CO-H, and CO-N groups displayed as x-fold induction values relative to the CO-N (control normoxic) group. Data were collected from three independent experiments (*** *p* < 0.001; **** *p* < 0.0001).

To investigate the angiogenic potential of the treated and untreated iMSCs, the angiogenesis-related genes in iMSCs were analyzed. It is well known that interleukin-(IL-)-8 and VEGF-A are critical regulators of angiogenesis [33,34]. In our present study, it was evident that the IL-8 gene expression in iMSCs from the IFN-H group (IFN-H: 102.77 ± 1.89) was significantly higher than in the other three groups (IFN-N group: 7.26 ± 0.24, CO-H group: 1.36 ± 0.13 and CO-N group: 1 ± 0.25), and the expression of the IL-8 gene in iMSCs from the IFN-N group was also significantly higher than the IL-8 levels from the CO-H and CO-N groups (Figure 3A). Concerning the VEGF-A gene expression, the levels in the IFN-H and IFN-N groups (IFN-H: 3.93 ± 0.12; IFN-N: 4.31 ± 0.11) were found to be significantly higher compared to those detected in the CO-H and CO-N groups (CO-H: 0.75 ± 0.25; CO-N: 1 ± 0.22). No significant differences were detected between the stimulated and unstimulated groups (Figure 3B). In summary, these results indicate that with IFN-γ pre-conditioned iMSCs possess a higher angiogenic potential compared to the control iMSCs.

### 3.3. Examination of IL-8 and VEGF-A Protein Concentrations in Pre-Conditioned iMSCs Secretomes

**Figure 4:**
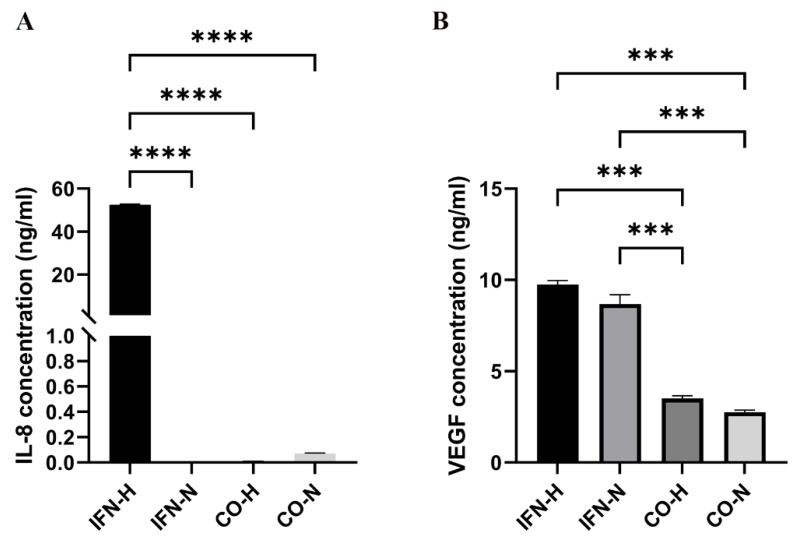
Quantification of IL-8 (**A**) and VEGF (**B**) protein concentration in secretomes from iMSCs pre-conditioned with IFN-γ under hypoxic and normoxic conditions. Values represent means ± SEM values from three independent experiments (*** *p* < 0.001; **** *p* < 0.0001).

To quantify the IL-8 and VEGF protein release in secretomes from different pre-conditioned iMSCs groups, we used human IL-8 and human VEGF ELISA kits. As shown in Figure 4, VEGF secretion was significantly increased in both IFN-stimulated groups (IFN-H: 9.75 ± 0.22; IFN-N: 8.68 ± 0.50) compared to levels detected in the unstimulated groups (CO-H: 3.51 ± 0.14; CO-N: 2.75 ± 0.11). After quantification of IL-8 protein secretion, a significant upregulation was detected in the IFN-H group (IFN-H: 52.48 ± 0.30) compared to the normoxic and untreated groups (IFN-N: 0.002 ± 0.001; CO-H: 0.01 ± 0.003; CO-N: 0.07 ± 0.004).

### 3.4. Tube Formation Assays with HUVECs Cultured in the Presence of Secretomes from Pre-Conditioned iMSCs

**Figure 5:**
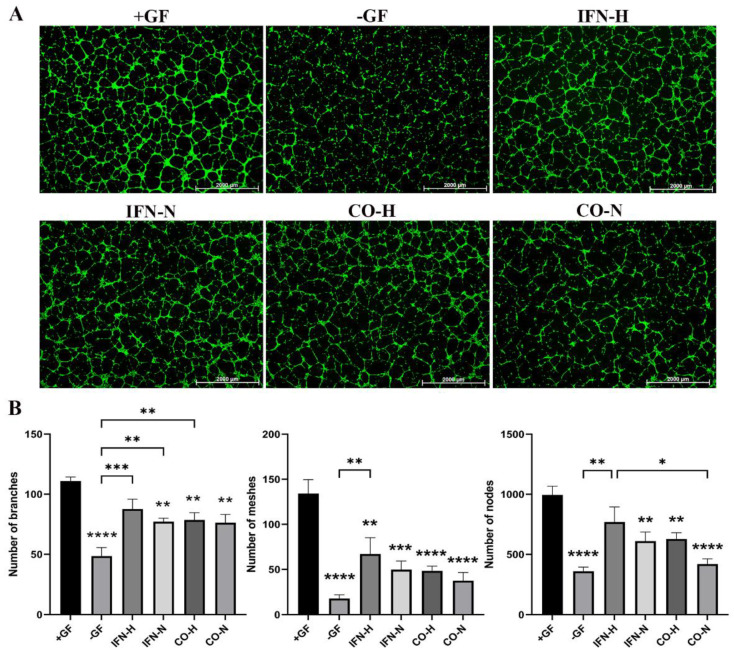
Tube formation of HUVECs incubated with secretomes obtained from differently pre-conditioned iMSCs. HUVECs seeded onto the Geltrex matrix in a medium containing secretomes from differently pre-conditioned iMSCs, growth factors (+GF, positive control group), or without growth factors (−GF, negative control group). (**A**) Representative images (1.25× magnification, scale bar = 2000 µm) of tube formation were taken using fluorescent microscopy (calcein-AM green staining), 8 h after cell seeding. (**B**) Number of branches, number of meshes, and number of nodes were quantified with the ImageJ software. Values represent means ±SEM from three independent experiments (*, **, ***, **** without bar indicates significant differences to the +GF group; * *p* < 0.05, ** *p* < 0.01, *** *p* < 0.001, **** *p* < 0.0001).

In order to evaluate the angiogenic potential of the secretomes obtained from differently pre-conditioned iMSCs, endothelial tube formation assays were performed. After imaging, different amounts of tube-like structures were formed. For the quantification using the Image J software, we used three indicators to determine the angiogenic effects of the different iMSC secretomes: the number of nodes, number of meshes, and the number of branches. As illustrated in Figure 5, HUVECs from the positive group (+GF) formed significantly more nodes, meshes, and branches (Figure 5B) than the cells from all other groups, except the number of branches and nodes compared to HUVECs from the IFN-H group. HUVECs from the IFN-H group formed significantly higher numbers of nodes, branches, and meshes compared to the cells from the negative control group (−GF) and in the tendency compared to all other groups, without reaching statistical significance.

### 3.5. Wound Healing Assays with HUVECs Cultured in the Presence of Pre-Conditioned iMSCs Secretomes

**Figure 6:**
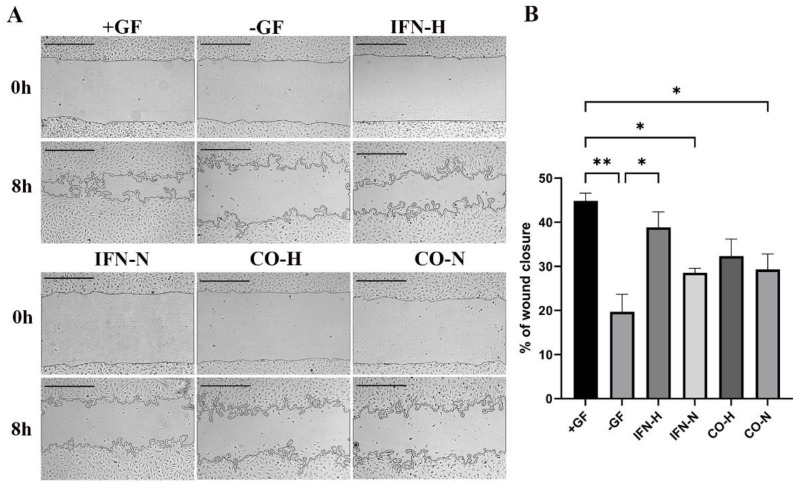
Wound healing assay of HUVECs incubated with differently pre-conditioned iMSCs secretomes. HUVECs cultured with differently pre-conditioned iMSCs secretomes, growth factors (+GF, positive group), or without growth factors (−GF, negative control group). (**A**) Representative images (5× magnification, scale bar = 500 µm) of the scratched area were taken using brightfield microscopy at 0 and 8 h after cell seeding. (**B**) Quantification of the wound closure by HUVECs cultured with pre-conditioned iMSCs secretomes. Values represent means ± SEM from three independent experiments (* *p* < 0.05, ** *p* < 0.01).

The migration capability of the endothelial cells represents the first step in the angiogenesis process [35]. In order to evaluate the wound closure by the migration capacity of HUVECs cultured in the presence of pre-conditioned iMSCs secretomes, wound healing assays were performed. After imaging, the wound closure area in the different groups was calculated by ImageJ. As shown in Figure 6, the wound closure percentage of the positive cell group (+GF: 44.83 ± 1.77%) was significantly higher (Figure 6B) than in the other groups (−GF: 19.68 ± 3.97%; IFN-H: 38.82 ± 3.50%; IFN-N: 28.54 ± 1.02%; CO-H: 32.31 ± 3.86%; CO-N: 29.27 ± 3.55%), except when compared to cells from the IFN-H group. HUVECs from the IFN-H group showed the highest percentages of wound closure, which reached statistical significance compared to the negative control group, which was cultured in the absence of all growth factors (−GF).

### 3.6. Gene Expression Analysis of Angiogenic Markers by HUVECs Cultured in the Presence of Pre-Conditioned iMSC Secretomes

**Figure 7:**
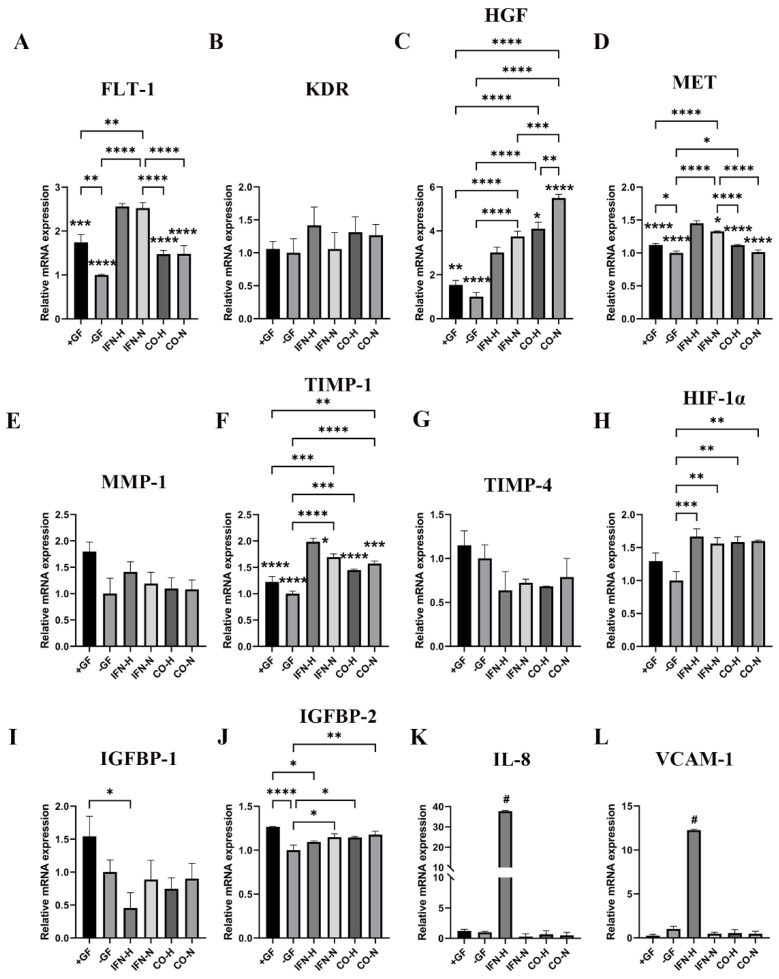
Expression of angiogenesis-related genes by HUVECs cultivated in the presence of pre-conditioned iMSCs secretomes. Gene expression levels of FLT-1 (**A**), KDR (**B**), HGF (**C**), MET (**D**), MMP-1 (**E**), TIMP-1 (**F**), TIMP-4 (**G**), HIF-1α (**H**), IGFBP-1 (**I**), IGFBP-2 (**J**), IL-8 (**K**), and VCAM-1 (**L**) in HUVECs cultivated in the presence of pre-conditioned iMSCs secretomes. Quantification of mRNA levels was performed using a Thermo Fisher Scientific PCR instrument, and ratios of the target mRNA copy numbers to copy numbers of the housekeeping gene (GAPDH) were calculated in all samples. Mean values ± SEM in HUVECs from different iMSCs secretome groups are displayed as x-fold induction values relative to the negative control group (−GF). Data were collected from three independent experiments (*, **, ***, **** without bar indicates significant differences compared to the IFN-H group, # indicates that the IFN-H group show significant differences to all other groups, * *p* < 0.05; ** *p* < 0.01; *** *p* < 0.001; **** *p* < 0.0001).

Further investigation of the biofunctionality of the differently pre-conditioned iMSCs secretomes includes the analysis of their effects on the angiogenic gene expression in HUVECs (Figure 6). HUVECs cultured in the presence of iMSC secretome from the IFN-γ (for 5 days) stimulation under hypoxic condition group (IFN-H) showed an up-regulation of seven pro-angiogenic genes (FLT-1, KDR, MET, TIMP-1, HIF-1α, IL-8, and VCAM-1) and a down-regulation of two anti-angiogenic genes (TIMP-4 and IGFBP-1) when compared to all other groups. The MMP-1 gene expression in the IFN-H HUVEC group was higher than that detected in the IFN-N, CO-H, CO-N, and −GF groups and lower than that of the +GF group, however, there was no significant difference among these groups (Figure 7E). The gene expression of IGFBP-2 in the IFN-H, IFN-N, CO-H, and CO-N groups was higher than in the −GF group and lower than that of the +GF HUVEC group (Figure 7J). Concerning the expression of the HGF gene, higher mRNA levels were detected in the IFN-H group compared to those detected in the +GF and -GF groups, but they were lower compared to those of the IFN-N, CO-H, and CO-N groups. The statistical significances are illustrated in Figure 7. The expression pattern of the 12 analyzed genes gives an overview of the iMSC secretome with the highest angiogenic potential under hypoxic and pro-inflammatory (IFN-γ) pre-treatment.

### 3.7. Sprouting Assays by HUVEC Spheroids Seeded on 3D Collagen/Hydroxyapatite Composites in the Presence of Pre-conditioned iMSC Secretomes

**Figure 8:**
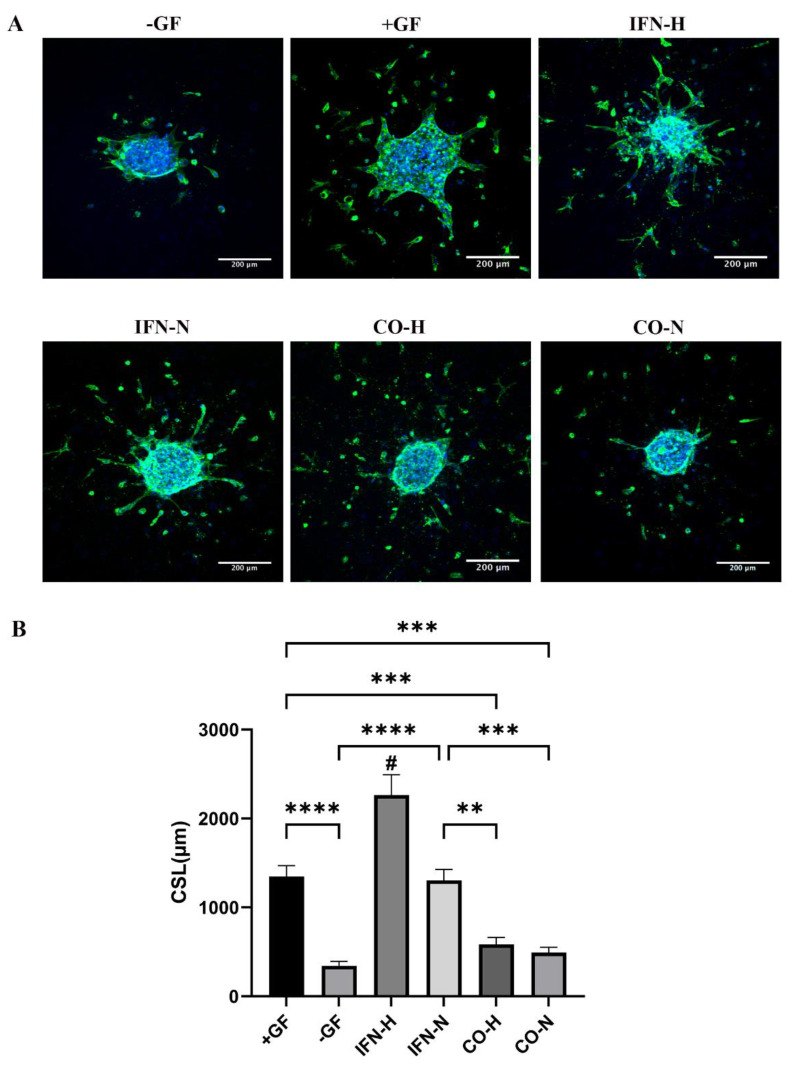
Sprout formation by 3D HUVEC spheroids cultured within coll/HA composites in the presence of iMSC secretomes for 48 h. (**A**) Representative confocal images (Alexa488-Phalloidin staining) of HUVEC spheroids cultured within coll/HA composites in the presence of differently pre-conditioned iMSCs secretomes in a 10× magnification (scale bar = 200 µm). (**B**) Quantification of cumulative sprouts length (CSL) analyzed using the ImageJ software. Mean CSL was calculated for at least 10 randomly selected spheroids per experimental group. Values represent means ± SEM from three independent experiments (# indicates that the IFN-H HUVEC group showed significant differences to all other groups, ** *p* < 0.01; *** *p* < 0.001; **** *p* < 0.0001).

To evaluate the functionality of differently conditioned iMSCs secretomes also in the 3D-culture, sprouting assays with HUVEC spheroids were performed within collagen/hydroxyapatite (coll/HA) composite scaffolds. We described the generation of these scaffolds in a previous work [36]. HUVEC spheroids were incorporated into coll/HA scaffolds and incubated in the presence of differently pre-conditioned iMSCs secretomes (IFN-H, IFN-N, CO-H, and CO-N groups). Figure 8 gives an overview of the merged images; the respective cytoplasmic and nuclear staining is illustrated in the Supplementary Figure S3. Sprout lengths were analyzed by ImageJ software after 48 h of incubation (Figure 8A). By quantifying the cumulative sprout length (CSL), we detected a significantly higher CSL in the IFN-H scaffold group (IFN-H: 2263.25 ± 228.68), compared to the CSL detected for all other groups (+GF: 1347.29 ± 122.98; −GF: 342.35 ± 49.52; IFN-N: 1303.20 ± 122.75; CO-H: 584.57 ± 76.12; CO-N: 492.75 ± 57.85). The CSL calculated in the scaffolds from the IFN-N and the +GF groups were significantly higher than those detected in the scaffolds from the CO-H, CO-N, and −GF groups. (Figure 8B). The CSL calculated in scaffolds from the CO-H and CO-N groups was higher than that calculated for the −GF group; however, without statistical significance.

## 4. Discussion

MSCs have been widely explored for cell-based therapy in the field of tissue regeneration due to their remarkable immunosuppressive and immunomodulatory properties [37], and their ability to enhance angiogenesis and accelerate tissue healing [38]. The similarity of iMSCs to primary MSCs and their regenerative potential in vivo have already been demonstrated in initial studies [39,40], but further investigation is needed. MSC-derived secretomes contain many bioactive molecules, such as growth factors, cytokines, chemokines, free nucleic acids, lipids, and extracellular vesicles, which carry proteins and/or miRNAs to target cells [41,42,43]. Detailed transcriptome analysis of iMSCs showed a rejuvenation-associated gene signature, as well as more genes in common with fetal MSCs than with adult MSCs [20]. The same study demonstrated that protein composition of iMSC secretomes is similar to that of both fetal and adult MSCs. However, the secretome composition can be regulated by preconditioning strategies during the in vitro iMSC culture [44]. The influence of different conditions has been investigated so far, including hypoxic and inflammatory conditions, addition of pharmacological agents, and 3D culture conditions [45]. A recent study investigated the influence of interferon-γ and hypoxia on the proteome and metabolome of therapeutic adipose-derived mesenchymal stem cells [46]. Pro-inflammatory and hypoxic conditions coexist in settings of chronic diseases, acute injury, and adipositas. The authors demonstrated that dual priming (IFN-γ/hypoxia) of MSCs intensified their immunomodulatory capacity, promoted their own survival, prevented them from clearance, and led to an anti-fibrotic MSC phenotype. Hypoxic conditions switch MSCs to glycolysis, causing fast consumption of glucose and fast production of lactate, which has inhibitory effects on T-cells. For the manufacturing of clinical-grade MSCs, there is a need to develop standardized assays to prove their potency. Guan and co-authors [47], detected after IFN exposure of MSCs, elevated expression levels of IDO and PD-L1 (programmed death ligand 1), which correlated with their suppressive potential on third-party T cell proliferation. The authors concluded that flow cytometric measurement of intracellular IDO and cell surface protein PD-L1 represents a potential and rapid assay for the assessment of their immunosuppressive potential.

Far less is known about the phenotype and features of iMSCs, as well as about their therapeutic capacities. Our study shows for the first time the effects of a pro-inflammatory IFN-γ activation under a hypoxic condition (5% O_2_) on the angiogenic potential of the obtained iMSCs secretome. The obtained angiogenic potency was compared to that induced by the normoxic (20% O_2_) and non-inflammatory environment of in vitro cultured iMSCs.

Pro-angiogenic proteins secreted by MSCs are mediated by growth factors (such as VEGF) and chemokines (such as IL-8) [48]. Many studies report that MSC secretomes can exert different effects in the context of angiogenesis, and many of these differences largely depend on the preconditioning of MSC cultures [49]. By the results described in the present study, we completely agree with this finding. Furthermore, the tissue origin seems to also influence the angiogenic profile of the human MSC secretome [50]. The use of rejuvenated iMSCs could bypass tissue- and age-related heterogeneities, which are associated with primary MSCs. In our study, we demonstrate for the first time that pro-inflammatory and hypoxic conditions enhance the angiogenic potency of the released iMSC secretome. As IL-8 and VEGF are both potent regulators of angiogenesis [33,34], we analyzed the gene and protein expression of both factors and detected the highest levels in the IFN-γ and hypoxia pre-conditioned iMSC group.

In recent years, priming approaches have been investigated to empower MSC efficacy. Among them, preconditioning with IFN-γ under hypoxic conditions seems to enhance the immunosuppressive properties of MSCs [45]. Under IFN-γ priming, MSCs increase the expression of class II histocompatibility leukocyte antigen (HLA) molecules [51], and MCSs preconditioning with IFN-γ and TNF-α in combination promoted angiogenesis and accelerated tumor growth [52]. Under hypoxic conditions, MSCs have been shown to possess a higher angiogenic and regenerative potential than under normoxic conditions [53]. This result is exactly in line with our obtained findings. Exosomes released by hypoxia-treated adipose tissue derived MSCs have been shown to enhance angiogenesis through the protein kinase A (PKA) signaling pathway in HUVECs [54]. Low oxygen tension is thought to be an integral component of the endosteal niche microenvironment. As IL-8 secretion by human primary MSCs is clearly increased under hypoxic conditions and IL-8 in turn possesses strong pro-angiogenic and chemotactic abilities, MSCs can enhance their migratory capacity in an autocrine manner [55] and promote osteogenesis at the same time [56]. However, IFN-γ exposure alone led to the suppression of VEGF and IL-8 by adipose-derived stromal cells. Conditioned medium of IFN-γ primed ASCs was not able to activate in vivo vessel formation [57]. Dual primed MSCs downregulated thrombospondin 1 and 2 expression, both factors that inhibit angiogenesis [46]. We did not analyze the thrombospondin expression, but its suppression might be a reason for the pro-angiogenic effect of the iMSC secretome from the IFN-H group, in addition to the suppression of the anti-angiogenic TIMP-4 and IGFBP-1, as described below.

Currently, we developed collagen/hydroxyapatite composites with good angiogenic properties by incorporating VEGF-mimetic peptides [36]. However, a cell type with pro-angiogenic properties could further support construct endothelialization in situ. Due to the limited proliferation and differentiation capacity of the primary jaw periosteal cells we usually work with, we found with iMSCs an alternative cell source that we derived from reprogrammed JPCs. As this MSC-like cell type is new, we drew our attention to a detailed analysis of its phenotype and functions, and demonstrated that iMSC priming with IFN-γ under hypoxic conditions induced pro-angiogenic properties of their secretome.

Besides evaluating the effect of iMSCs secretome on HUVEC tube formation, we also investigated the angiogenic gene expression in HUVECs. The analyzed genes play an important role during angiogenesis. VEGFR-1 (FLT-1) and VEGFR-2 (KDR) are both receptors of VEGF, representing principal initiators of the angiogenesis process [58]. For angiogenesis amplification and vascular stabilization, other factors like IL-8, IGFBP-2, MMP-1, VCAM-1, HIF-1α, HGF, and MET come into play [59,60,61,62,63]. Angiogenesis inhibitory factors like TIMP-4, endostatin, angiostatin, or thrombospondin suppress vascular growth [64]. Although several studies demonstrated that the inhibition of TIMP1 promotes angiogenesis [65], there are also contradictory works reporting on enhanced tumor angiogenesis in the brain metastasis of lung carcinoma associated with TIMP-1 overexpression [66]. In our study, HUVECs cultured in the presence of IFN-γ and hypoxia pre-conditioned iMSCs secretome showed an up-regulation of pro-angiogenic genes (FLT-1, KDR, MET, TIMP-1, HIF-1α, IL-8, and VCAM-1) and a down-regulation of anti-angiogenic genes such as TIMP-4, IGFBP-1, and IGFBP-2, compared to all other groups. Relatively little is actually known about the function of TIMP-4 and its role in angiogenesis. However, TIMP-4 has been shown to inhibit platelet aggregation and to decrease the migration and invasive potential of cancer cell lines. Further studies could demonstrate the inhibitory effect of TIMP-4 on capillary tube formation only in high doses and on capillary endothelial cell migration [67]. We suggest that TIMP-4 inhibition in the HUVEC group cultivated with the pre-conditioned (IFN-γ and hypoxia) iMSCs secretome was based on its anti-angiogenic effect. IGFBPs usually inhibit the metabolic and proliferative actions of IGFs by binding them, prolonging their half-lives, and altering their interactions with cell surface receptors. Hypoxia induces IGFBP-1 hyperphosphorylation, leading to decreased IGF-I bioavailability in fetal growth [68]. However, the interactions between IGFs and IGFBPs are mutual, resulting in reciprocal regulation dependent on the cellular environment [66].

Additionally, the HGF gene expression has been shown to be inversely proportional to detected c-MET levels in the pre-conditioned iMSCs secretome HUVEC group, compared to the other groups. HGF unfolds its angiogenic effect via tyrosine phosphorylation of its specific receptor, c-Met, expressed on blood vessels including endothelial cells (ECs) and vascular smooth muscle cells (VSMCs) [69]. Based on maximal c-MET transcription levels detected in the IFN-H group, we suppose higher HGF-responsiveness in this group compared to the other IFN-treated or untreated groups. The pre-conditioned iMSCs secretome obtained upon IFN-γ and hypoxia treatment could also include TGF-beta [51], which is able to inhibit the expression of HGF [70]. We suppose this might be the reason for reduced HGF levels in the IFN-H group compared to the other three secretome groups.

Many studies have shown the relevance of VCAM-1 in angiogenesis [71], and the chemokine interleukin-8 exerts potent pro-angiogenic effects through binding to the CXCR2 receptor of intestinal microvascular endothelial cells and downstream signaling by phosphorylation of the extracellular signal-regulated protein kinase 1/2 (ERK 1/2) [72]. As IL-8 and VCAM-1 gene expressions have been shown to be strongly and significantly upregulated in treated HUVECs, we suggest that these are key responder factors induced by the strong pro-angiogenic effect starting from the IFN-γ/hypoxia pre-conditioned iMSC secretome. We did not analyze the gene expression pattern of 3D-cultured HUVECs (as shown in Figure 8), but sprout formation assays showed very clearly similar results to the performed tube formation assays with 2D-cultured HUVECs (as shown in Figure 5). These results showed that regardless of the cultivation approach in 2D on a standard growth-factor reduced matrigel preparation composed of laminin I, type IV collagen, entactin, and heparan sulfate proteoglycans (information from the company), or in 3D within the type I collagen/hydroxyapatite constructs, HUVECs were able to be activated by the IFN-γ and hypoxia pre-conditioned iMSCs secretome.

Taken together, in this study we provide insight into the excellent pro-angiogenic functional capacity starting from IFN-γ and hypoxia pre-conditioned iMSCs secretome.

## 5. Conclusions

In this study, we present a cell-free approach for the development of collagen/hydroxyapatite scaffolds exhibiting angiogenic properties. Secondly, we demonstrate that dual priming with hypoxia and IFN-γ significantly improved the pro-angiogenic properties of iMSCs. Based on this result, we conclude that iMSCs priming before clinical application can activate neovascularization and improve the therapeutic efficacy of these stem cells.

## Figures and Tables

**Table 1:** List of the antibodies used for the detection of MSC and iPSC marker expression on iMSCs; * Biolegend, ** Miltenyi. Rec-recombinant.

	PE-Labeled Antibodies	Volume [µL]	APC-Labeled Antibodies	Volume [µL]
1	Rec. Tra-1–0	1 **	CD90 (IgG1)	5 *
2	Rec. Tra-1–81	1 **	CD73 (IgG1)	5 *
3	Rec. SSEA4	5 **	CD105 (IgG1)	5 *
4	CD44 (IgG1)	5 *	CD326 (IgG2a *)	5 *
5	Rec.-PE (control)	5 *	IgG2a-APC (control)	5 *
6	IgG1-PE (control)	5 **	IgG1-APC (control)	5 *

## Data Availability

Obtained data for this study are available from the corresponding author upon reasonable request.

## Supplementary Materials

The following supporting information can be downloaded at: https://www.mdpi.com/article/10.3390/cells11060988/s1, Figure S1: Representative histograms of MSC- and iPSC-surface marker expression on iMSCs of passage 3. Figure S2: Tube formation of HUVECs incubated with different concentrations of iMSCs secretome; Figure S3: Sprout formation by 3D HUVEC spheroids cultured within coll/HA composites for 48 h.
