# Supplementary material for: Pre-Conditioning with IFN-γ and Hypoxia Enhances the Angiogenic Potential of iPSC-Derived MSC Secretome
Source: Cells. 2022 Mar 14;11(6):988. doi: 10.3390/cells11060988 (PMC8946902; doi:10.3390/cells11060988)
Supplement: Supplementary file 1 [file cells-11-00988-s001.zip › cells-1614349-supplementary.pdf]

Supplementary figures

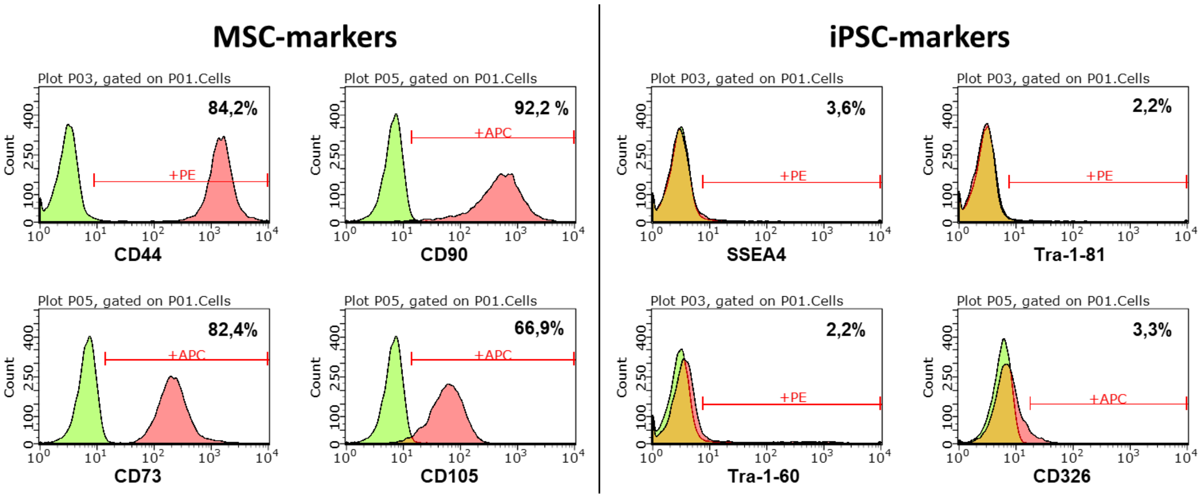

Fig. S1 Representative histograms of MSC- and iPSC-surface marker expression by iMSCs of passage 3 (green = isotype control, red = surface marker). iMSCs show high expression of MSC-markers while iPSC-marker expression is downregulated.

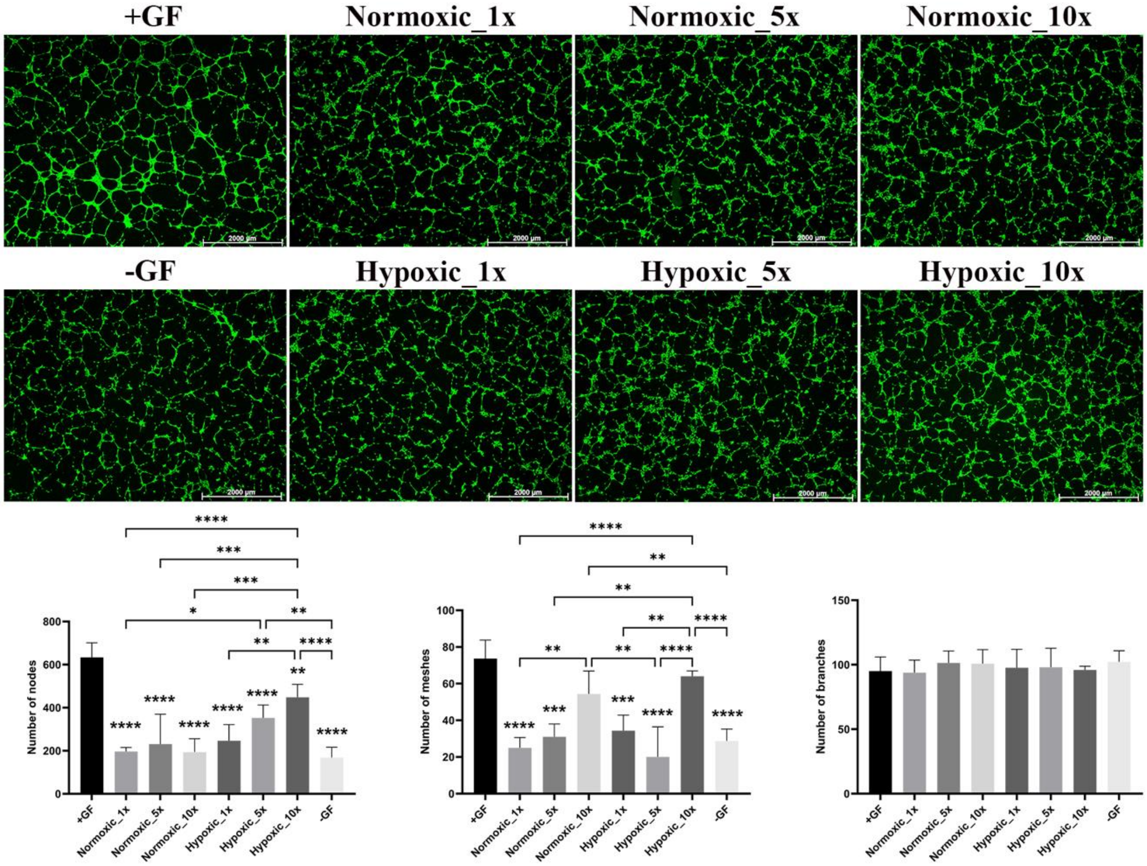

Fig. S2 Tube formation of HUVECs incubated with different concentrations of iMSCs secretome. HUVECs seeded on Geltrex matrix in medium containing different concentrations of iMSCs secretome, +GF (positive group), or without growth factors (-GF, negative group). Number of branches, number of meshes, and number of nodes were quantified with the ImageJ software. Values represent means ± SEM from 3 independent experiments (*, **, ***, **** without bar indicates significant differences to the +GF group; * $p < 0.05$, ** $p < 0.01$, *** $p < 0.001$, **** $p < 0.0001$).

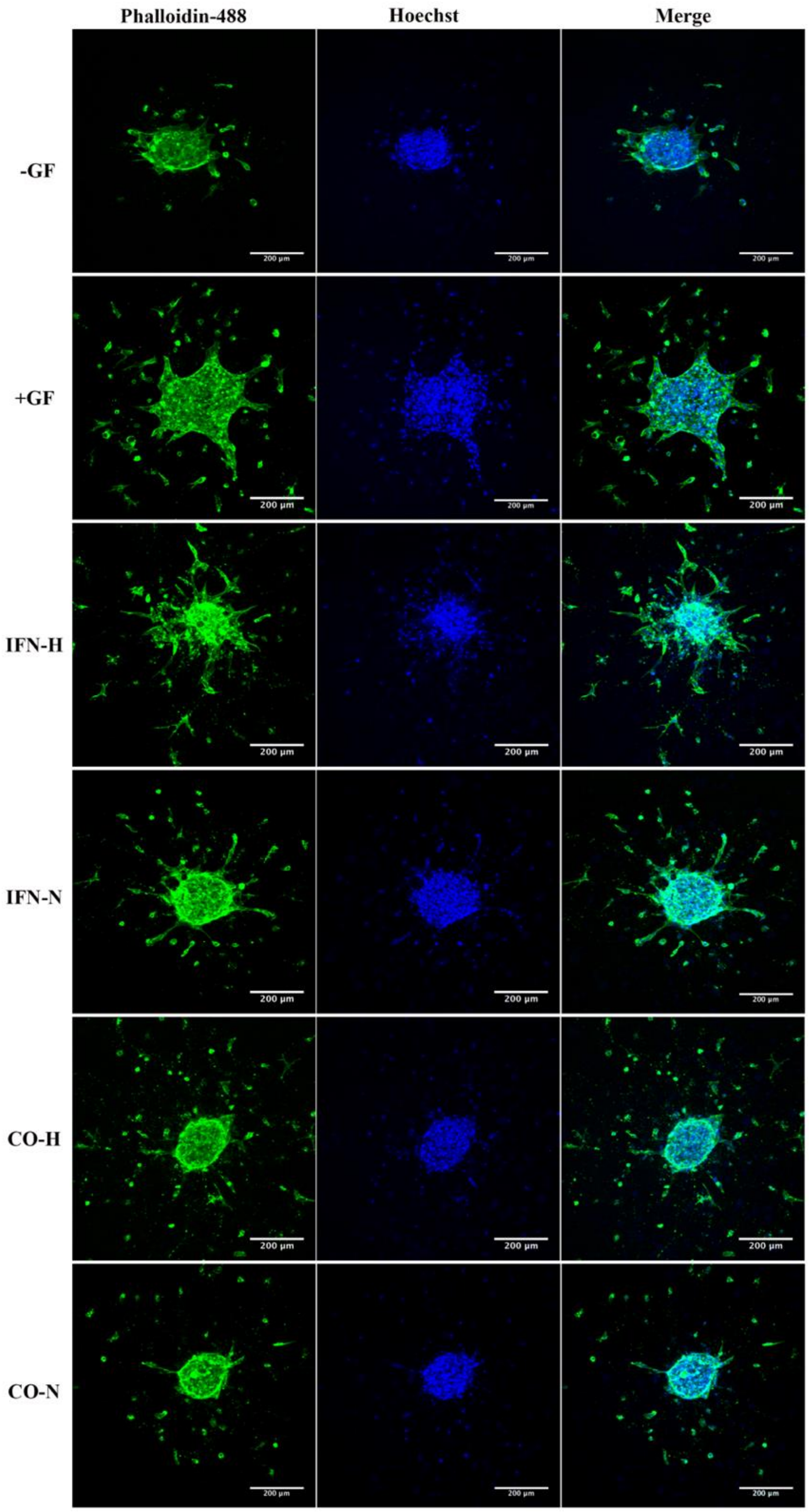

Fig. S3 Sprout formation by 3D HUVEC spheroids cultured within coll/HA composites for 48 hours. Representative confocal microscopic images (Alexa488-

Phalloidin staining) of HUVEC spheroids cultured within coll/HA composites comprising different conditioned iMSCs secretome in a 10x magnification (scale bar = 200 µm).
